# (2,7-Dimeth­oxy­naphthalen-1-yl)(phen­yl)methanone

**DOI:** 10.1107/S1600536810038195

**Published:** 2010-09-30

**Authors:** Yuichi Kato, Atsushi Nagasawa, Daichi Hijikata, Akiko Okamoto, Noriyuki Yonezawa

**Affiliations:** a Department of Organic and Polymer Materials Chemistry, Tokyo University of Agriculture & Technology, Koganei, Tokyo 184-8588, Japan

## Abstract

The asymmetric unit of the title compound, C_19_H_16_O_3_, contains three independent conformers. Each of the three conformers has essentially the same feature of non-coplanar aromatic rings whereby the aroyl group at the 1-position of the naphthalene ring is twisted in a perpendicular manner to the naphthalene ring. The dihedral angles between the benzene ring planes and the naphthalene ring systems are 75.34 (7), 86.47 (7) and 76.55 (6)° in the three conformers. The crystal structure is stabilized by inter­molecular C—H⋯O hydrogen bonds.

## Related literature

For electrophilic aromatic substitution of naphthalene deriva­tives, see: Okamoto & Yonezawa (2009 ▶). For the structures of closely related compounds, see: Nakaema, Watanabe *et al.* (2008 ▶); Mitsui *et al.* (2008 ▶); Watanabe, Nagasawa *et al.* (2010 ▶); Hijikata, Nakaema, Watanabe *et al.* (2010*a* ▶,*b* ▶).
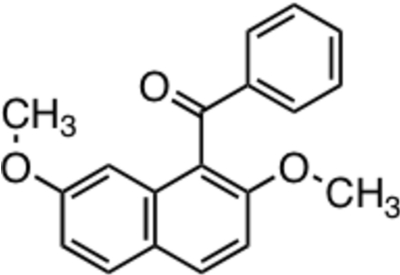


## Experimental

### 

#### Crystal data

C_19_H_16_O_3_
*M* *_r_* = 292.32
Monoclinic, 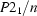

*a* = 23.4356 (4) Å
*b* = 7.84115 (14) Å
*c* = 26.7438 (5) Å
β = 111.786 (1)°
*V* = 4563.49 (14) Å^3^
*Z* = 12
Cu *K*α radiation
μ = 0.69 mm^−1^
*T* = 193 K
0.60 × 0.20 × 0.10 mm

#### Data collection

Rigaku R-AXIS RAPID diffractometer
Absorption correction: numerical (*NUMABS*; Higashi, 1999 ▶) *T*_min_ = 0.682, *T*_max_ = 0.934
76914 measured reflections
8353 independent reflections
6698 reflections with *I* > 2σ(*I*)
*R*_int_ = 0.021

#### Refinement

*R*[*F* ^2^ > 2σ(*F* ^2^)] = 0.039
*wR*(*F* ^2^) = 0.109
*S* = 1.12
8353 reflections
602 parameters
H-atom parameters constrained
Δρ_max_ = 0.22 e Å^−3^
Δρ_min_ = −0.16 e Å^−3^

### 

Data collection: *PROCESS-AUTO* (Rigaku, 1998 ▶); cell refinement: *PROCESS-AUTO*; data reduction: *CrystalStructure* (Rigaku/MSC, 2004 ▶); program(s) used to solve structure: *SIR2004* (Burla *et al.*, 2005 ▶); program(s) used to refine structure: *SHELXL97* (Sheldrick, 2008 ▶); molecular graphics: *ORTEPIII* (Burnett & Johnson, 1996 ▶); software used to prepare material for publication: *CrystalStructure*.

## Supplementary Material

Crystal structure: contains datablocks I, global. DOI: 10.1107/S1600536810038195/om2366sup1.cif

Structure factors: contains datablocks I. DOI: 10.1107/S1600536810038195/om2366Isup2.hkl

Additional supplementary materials:  crystallographic information; 3D view; checkCIF report

## Figures and Tables

**Table 1:** Hydrogen-bond geometry (Å, °)

*D*—H⋯*A*	*D*—H	H⋯*A*	*D*⋯*A*	*D*—H⋯*A*
C36—H36⋯O2	0.95	2.56	3.4116 (18)	149
C56—H56*A*⋯O5^i^	0.98	2.54	3.4862 (19)	161
C52—H52⋯O3^i^	0.95	2.46	3.395 (2)	168
C34—H34⋯O6^ii^	0.95	2.41	3.143 (2)	133
C54—H54⋯O9^ii^	0.95	2.58	3.2451 (19)	128
C19—H19*B*⋯O9^iii^	0.98	2.59	3.116 (2)	113

Symmetry codes: (i) 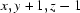; (ii) 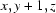; (iii) 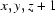.

**Fig. 1.**
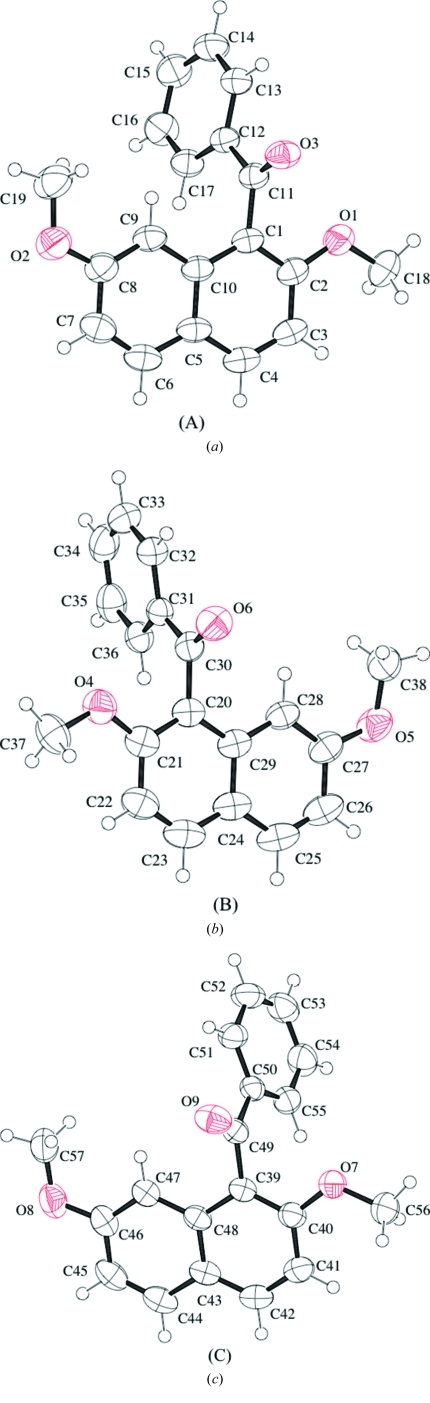
The structure of the conformers (A), (B), and (C), showing the atom labeling scheme. Displacement ellipsoids are drawn at the 50% probability level for non-H atoms.

**Fig. 2.**
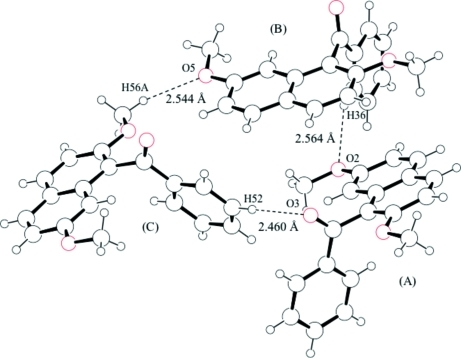
The intermolecular C—H···O hydrogen bonds among conformer (A), (B), and (C). They are shown as dashed lines.

**Fig. 3.**
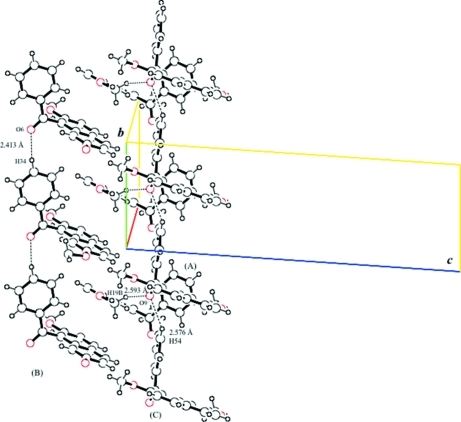
Partial crystal packing diagram, viewed down the a axis. C—H···O hydroghen bonds are shown as dashed lines.

**Fig. 4.**
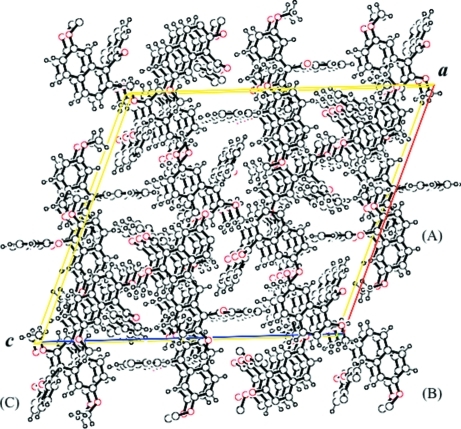
The arrangement of the molecules in the crystal structure, viewed down the b axis.

## supplementary crystallographic information

### Comment

In the course of our study on electrophilic aromatic aroylation of 2,7-dimethoxynaphthalene, *peri*-aroylnaphthalene compounds have proven to be formed regioselectively with the aid of suitable acidic mediators (Okamoto & Yonezawa, 2009). Recently, we reported the crystal structures of several 1,8-diaroylated naphthalene homologues exemplified by (2,7-dimethoxynaphthalene-1,8-diyl)bis(4-fluorobenzoyl)dimethanone (Watanabe *et al.*, 2010). The aroyl groups at the 1,8-positions of the naphthalene rings in these compounds are connected almost perpendicularly but the benzene ring moieties of the aroyl groups tilt slightly toward the *exo* sides of the naphthalene rings. In the crystal of 1,8-dibenzoyl-2,7-dimethoxynaphthalene (Nakaema *et al.*, 2008), molecules are arranged by C–H···O hydrogen bonding along the *c* axis of the unit cell, and a π–π stacking interaction perpendicular to the *bc* plane is also observed. Moreover, the X-ray crystal structural analyses of 1-(4-substituted benzoyl)naphthalenes, *i.e.*, 1-(4-chlorobenzoyl)-2,7-dimethoxynaphthalene (Mitsui *et al.*, 2008), 2-(2,7-dimethoxy-1-naphthoyl)benzoic acid (Hijikata *et al.*., 2010*a*) and methyl 4-(2,7-dimethoxy-1-naphthoyl)benzoate (Hijikata *et al.*, 2010*b*), have also revealed to have essentially the same non-coplanar structure with the 1,8-diaroylated naphthalenes. As a part of the course of our continuous study on the molecular structures of this kind of homologous molecules, the crystal structure of title compound, 1-benzoylatednaphthalene, is discussed in this paper.

There are three independent conformers in the crystal structure of the title compound. The independent conformers are labeled (A), (B), and (C) and are shown in Fig. 1. Each conformer has essentially the same non-coplanar structure. The respective dihedral angles between the naphthalene rings and the benzene rings of the three conformers are 75.34 (7), 86.46 (7), and 76.55 (6)°. The bridging carbonyl planes make large dihedral angles of 77.57 (7)° [C2—C1—C11—O3 torsion angle = -77.82 (18)°], 88.38 (7)° [C21—C20—C30—O6 torsion angle = -90.52 (18)°], and 81.87 (7)° [C40—C39—C49—O9 torsion angle = -101.94 (17)°] with the naphthalene ring systems. On the other hand, the dihedral angles between the bridging carbonyl planes and the benzene rings are rather small, such as 8.27 (9)° [O3—C11—C12—C17 torsion angle = -173.33 (14)°], 10.23 (9)° [O6—C30—C31—C36 torsion angle = -170.51 (15)°], and 15.95 (8)° [O9—C49—C50—C55 torsion angle = 167.01 (14)°]. The methyl groups of the methoxy groups adjacent to the aroyl groups, are oriented to the *exo* sites of the molecules and the other methyl groups are directed to *endo* sites. The crystal structure is stabilized by intermolecular hydrogen bonds among three different conformers and between same types of conformers (Table 1, Fig. 2, 3). Among three different conformers, hydrogen bonds between conformers (A) and (B) [C36–H36···O2= 2.564 Å], between conformers (B) and (C) [C56—H56A···O5 = 2.544 Å], and between conformers (C) and (A) [C52—H52···O3 = 2.460 Å] are observed. In addition, the conformers (B) are connected to each other by intermolecular hydrogen bonds between carbonyl oxygen and aromatic hydrogen [C34–H34···O6 = 2.413 Å].The conformers (C) are also linked with intermolecular hydrogen bonds between carbonyl oxygen and aromatic hydrogen [C54–H54···O9 = 2.576 Å]. The conformers (B) and (C) are stacked along the *b* axis and form the columnar structures, respectively. On the other hand, the conformers (A) have no interactions with themselves along the *b* axis. They have only weak intermolecular interactions with conformers (C) [C19–H19B···O9 = 2.593 Å] and are piled in the gap of the two different columnar structures of conformers (B) and (C) (Fig. 4).

### Experimental

To a 100 ml flask, benzoyl chloride (8.1 mmol, 0.923 ml), aluminium chloride (AlCl_3_; 10.4 mmo1, 1.38 g) and methylene chloride (CH_2_Cl_2_; 19 ml) were placed and stirred at 273 K. To the reaction mixture thus obtained 2,7-dimethoxynaphthalene (7.5 mmol, 1.372 g) in methylene chloride (CH_2_Cl_2_; 19 ml) were added. After the reactionmixture was stirred at 273 K for 6 h, it was poured into ice-cold water (10 ml)and the mixture was extracted with CHCl_3_ (10 ml × 3). The combined extracts were washed with 2 *M* aqueous NaOH followed by washing with brine. The organic layers thus obtained were dried over anhydrous MgSO_4_. The solvent was removed under reduced pressure togive a cake (98% yield). The crude product was purified by recrystallization from hexane-chloroform. Yellow platelet single-crystals suitable for X-ray diffraction were obtained by crystallization from hexane-methylene chloride.

Spectroscopic Data:

^1^H NMR δ (300 MHz, CDCl_3_); 3.71 (3*H*, s), 3.78 (3*H*, s, *J* = 8.6 Hz), 6.79 (1*H*, d, *J* = 2.4 Hz), 7.01 (1*H*, dd, *J* = 8.7, 2.4 Hz), 7.16 (1*H*, d, *J* = 9.0 Hz), 7.43 (2*H*, t, *J* = 7.8 Hz), 7.57 (1*H*, t, *J* = 7.2 Hz), 7.72 (1*H*, d, *J* = 8.7 Hz), 7.72 (1*H*, d, *J* = 8.7 Hz), 7.84–7.89 (3*H*, m) p.p.m..

^13^C NMR δ (300 MHz, CDCl_3_); 55.09, 56.24, 102.02, 110.17, 117.01, 121.67,124.29, 128.48, 129.44, 129.62, 130.96, 132.98, 133.31, 137.98, 154.93, 158.77,198.07 p.p.m..

IR(KBr):1663.30 (C=O), 1626.66 (Ar) cm^-1^.

HRMS(*m/z*): [*M* + H]^+^ Calcd for C_19_H_17_O_3_, 293.3365; found, 293.1185.

m.p.= 358.5–362 K

### Refinement

All H atoms were found in a difference map and were subsequently refined as riding atoms, with C–H = 0.95 (aromatic) and 0.98 (methyl) Å, and with *U*_iso_(H) = 1.2*U*_eq_(C).

### Crystal data

**Table:**

C_19_H_16_O_3_	*F*(000) = 1848
*M**_r_* = 292.32	*D*_x_ = 1.276 Mg m^−^^3^
Monoclinic, *P*2_1_/*n*	Cu *K*α radiation, λ = 1.54187 Å
Hall symbol: -P 2yn	Cell parameters from 57393 reflections
*a* = 23.4356 (4) Å	θ = 3.2–68.2°
*b* = 7.84115 (14) Å	µ = 0.69 mm^−^^1^
*c* = 26.7438 (5) Å	*T* = 193 K
β = 111.786 (1)°	Plate, colorless
*V* = 4563.49 (14) Å^3^	0.60 × 0.20 × 0.10 mm
*Z* = 12	

### Data collection

**Table:**

Rigaku R-AXIS RAPID diffractometer	8353 independent reflections
Radiation source: rotating anode	6698 reflections with *I* > 2σ(*I*)
graphite	*R*_int_ = 0.021
Detector resolution: 10.00 pixels mm^-1^	θ_max_ = 68.2°, θ_min_ = 3.2°
ω scans	*h* = −28→28
Absorption correction: numerical (*NUMABS*; Higashi, 1999)	*k* = −9→9
*T*_min_ = 0.682, *T*_max_ = 0.934	*l* = −32→32
76914 measured reflections	

### Refinement

**Table:**

Refinement on *F*^2^	Secondary atom site location: difference Fourier map
Least-squares matrix: full	Hydrogen site location: inferred from neighbouring sites
*R*[*F*^2^ > 2σ(*F*^2^)] = 0.039	H-atom parameters constrained
*wR*(*F*^2^) = 0.109	*w* = 1/[σ^2^(*F*_o_^2^) + (0.0527*P*)^2^ + 0.663*P*] where *P* = (*F*_o_^2^ + 2*F*_c_^2^)/3
*S* = 1.12	(Δ/σ)_max_ = 0.001
8353 reflections	Δρ_max_ = 0.22 e Å^−^^3^
602 parameters	Δρ_min_ = −0.16 e Å^−^^3^
0 restraints	Extinction correction: *SHELXL97* (Sheldrick, 2008), Fc^*^=kFc[1+0.001xFc^2^λ^3^/sin(2θ)]^-1/4^
Primary atom site location: structure-invariant direct methods	Extinction coefficient: 0.00122 (8)

### Special details

**Table:**

Geometry. All e.s.d.'s (except the e.s.d. in the dihedral angle between two l.s. planes) are estimated using the full covariance matrix. The cell e.s.d.'s are taken into account individually in the estimation of e.s.d.'s in distances, angles and torsion angles; correlations between e.s.d.'s in cell parameters are only used when they are defined by crystal symmetry. An approximate (isotropic) treatment of cell e.s.d.'s is used for estimating e.s.d.'s involving l.s. planes.
Refinement. Refinement of *F*^2^ against ALL reflections. The weighted *R*-factor *wR* and goodness of fit *S* are based on *F*^2^, conventional *R*-factors *R* are based on *F*, with *F* set to zero for negative *F*^2^. The threshold expression of *F*^2^ > σ(*F*^2^) is used only for calculating *R*-factors(gt) *etc*. and is not relevant to the choice of reflections for refinement. *R*-factors based on *F*^2^ are statistically about twice as large as those based on *F*, and *R*- factors based on ALL data will be even larger.

### Fractional atomic coordinates and isotropic or equivalent isotropic displacement parameters (Å^2^)

**Table:**

	*x*	*y*	*z*	*U*_iso_*/*U*_eq_	
O1	0.51486 (4)	0.16611 (14)	1.04957 (4)	0.0587 (3)	
O2	0.20220 (5)	0.45515 (18)	0.91435 (4)	0.0722 (3)	
O3	0.39690 (5)	0.04069 (13)	1.06125 (4)	0.0560 (3)	
O4	0.22870 (5)	0.15222 (16)	0.76495 (4)	0.0637 (3)	
O5	0.00579 (5)	−0.09657 (16)	0.87951 (4)	0.0670 (3)	
O6	0.08863 (5)	0.01795 (14)	0.71223 (4)	0.0641 (3)	
O7	0.03065 (4)	0.71601 (15)	0.02183 (4)	0.0577 (3)	
O8	0.24498 (5)	0.39304 (15)	0.27448 (4)	0.0674 (3)	
O9	0.16772 (5)	0.50304 (14)	0.06449 (5)	0.0648 (3)	
C1	0.41405 (6)	0.25521 (18)	1.00606 (5)	0.0440 (3)	
C2	0.47190 (6)	0.23724 (18)	1.00467 (5)	0.0475 (3)	
C3	0.48429 (7)	0.2895 (2)	0.95928 (6)	0.0532 (4)	
H3	0.5240	0.2738	0.9583	0.064*	
C4	0.43877 (7)	0.3629 (2)	0.91685 (6)	0.0551 (4)	
H4	0.4474	0.3988	0.8865	0.066*	
C5	0.37912 (7)	0.38701 (19)	0.91685 (5)	0.0497 (3)	
C6	0.33197 (8)	0.4678 (2)	0.87400 (6)	0.0594 (4)	
H6	0.3403	0.5080	0.8439	0.071*	
C7	0.27523 (8)	0.4887 (2)	0.87511 (6)	0.0633 (4)	
H7	0.2443	0.5443	0.8460	0.076*	
C8	0.26156 (7)	0.4287 (2)	0.91910 (6)	0.0555 (4)	
C9	0.30564 (6)	0.3513 (2)	0.96189 (5)	0.0501 (3)	
H9	0.2959	0.3118	0.9914	0.060*	
C10	0.36631 (6)	0.32983 (18)	0.96222 (5)	0.0448 (3)	
C11	0.40345 (6)	0.19262 (19)	1.05519 (5)	0.0438 (3)	
C12	0.40215 (6)	0.32003 (18)	1.09617 (5)	0.0426 (3)	
C13	0.40109 (6)	0.2633 (2)	1.14507 (5)	0.0506 (3)	
H13	0.3999	0.1446	1.1516	0.061*	
C14	0.40184 (8)	0.3792 (2)	1.18399 (6)	0.0633 (4)	
H14	0.4016	0.3401	1.2176	0.076*	
C15	0.40288 (8)	0.5516 (2)	1.17444 (7)	0.0673 (4)	
H15	0.4033	0.6307	1.2015	0.081*	
C16	0.40328 (8)	0.6104 (2)	1.12605 (6)	0.0614 (4)	
H16	0.4036	0.7294	1.1195	0.074*	
C17	0.40328 (6)	0.49406 (19)	1.08707 (6)	0.0501 (3)	
H17	0.4041	0.5338	1.0538	0.060*	
C18	0.57707 (7)	0.1598 (2)	1.05318 (7)	0.0634 (4)	
H18A	0.6028	0.1091	1.0877	0.076*	
H18B	0.5915	0.2756	1.0507	0.076*	
H18C	0.5797	0.0904	1.0236	0.076*	
C19	0.18576 (8)	0.4047 (3)	0.95842 (7)	0.0807 (6)	
H19A	0.1441	0.4446	0.9524	0.097*	
H19B	0.2147	0.4547	0.9918	0.097*	
H19C	0.1872	0.2801	0.9614	0.097*	
C20	0.15701 (6)	0.06924 (18)	0.80110 (5)	0.0469 (3)	
C21	0.21794 (7)	0.0817 (2)	0.80740 (6)	0.0544 (4)	
C22	0.26461 (7)	0.0244 (2)	0.85476 (7)	0.0658 (4)	
H22	0.3065	0.0315	0.8585	0.079*	
C23	0.24920 (8)	−0.0414 (2)	0.89516 (7)	0.0707 (5)	
H23	0.2809	−0.0801	0.9271	0.085*	
C24	0.18744 (8)	−0.0538 (2)	0.89106 (6)	0.0591 (4)	
C25	0.17046 (9)	−0.1199 (2)	0.93278 (7)	0.0729 (5)	
H25	0.2017	−0.1580	0.9651	0.087*	
C26	0.11109 (9)	−0.1306 (2)	0.92800 (6)	0.0715 (5)	
H26	0.1010	−0.1749	0.9567	0.086*	
C27	0.06410 (8)	−0.0755 (2)	0.87998 (6)	0.0562 (4)	
C28	0.07786 (7)	−0.00943 (18)	0.83870 (5)	0.0484 (3)	
H28	0.0458	0.0285	0.8069	0.058*	
C29	0.14004 (7)	0.00297 (18)	0.84305 (5)	0.0479 (3)	
C30	0.10937 (6)	0.12284 (18)	0.74798 (5)	0.0446 (3)	
C31	0.08962 (6)	0.30293 (18)	0.74001 (5)	0.0430 (3)	
C32	0.05343 (7)	0.3591 (2)	0.68843 (6)	0.0576 (4)	
H32	0.0416	0.2817	0.6590	0.069*	
C33	0.03497 (8)	0.5264 (3)	0.68028 (8)	0.0740 (5)	
H33	0.0108	0.5648	0.6451	0.089*	
C34	0.05123 (8)	0.6386 (2)	0.72272 (9)	0.0743 (5)	
H34	0.0379	0.7539	0.7168	0.089*	
C35	0.08686 (7)	0.5846 (2)	0.77408 (8)	0.0621 (4)	
H35	0.0980	0.6627	0.8033	0.074*	
C36	0.10630 (6)	0.41692 (18)	0.78287 (6)	0.0475 (3)	
H36	0.1310	0.3797	0.8181	0.057*	
C37	0.29031 (7)	0.1551 (3)	0.76654 (7)	0.0699 (5)	
H37A	0.2911	0.2093	0.7338	0.084*	
H37B	0.3058	0.0380	0.7688	0.084*	
H37C	0.3164	0.2198	0.7981	0.084*	
C38	−0.04394 (8)	−0.0657 (2)	0.83044 (6)	0.0654 (4)	
H38A	−0.0824	−0.1006	0.8340	0.078*	
H38B	−0.0381	−0.1314	0.8015	0.078*	
H38C	−0.0458	0.0561	0.8218	0.078*	
C39	0.09947 (6)	0.61155 (17)	0.10299 (5)	0.0419 (3)	
C40	0.04007 (6)	0.65911 (19)	0.07253 (5)	0.0476 (3)	
C41	−0.00646 (7)	0.6483 (2)	0.09395 (6)	0.0569 (4)	
H41	−0.0474	0.6806	0.0727	0.068*	
C42	0.00761 (7)	0.5913 (2)	0.14520 (6)	0.0583 (4)	
H42	−0.0240	0.5848	0.1593	0.070*	
C43	0.06762 (7)	0.54153 (18)	0.17787 (6)	0.0487 (3)	
C44	0.08383 (8)	0.4859 (2)	0.23198 (6)	0.0576 (4)	
H44	0.0531	0.4809	0.2472	0.069*	
C45	0.14218 (8)	0.4399 (2)	0.26228 (6)	0.0593 (4)	
H45	0.1521	0.4049	0.2985	0.071*	
C46	0.18824 (7)	0.44396 (19)	0.24015 (6)	0.0522 (3)	
C47	0.17515 (6)	0.49750 (17)	0.18847 (5)	0.0459 (3)	
H47	0.2066	0.5004	0.1741	0.055*	
C48	0.11454 (6)	0.54875 (17)	0.15616 (5)	0.0424 (3)	
C49	0.14990 (6)	0.62845 (18)	0.08130 (5)	0.0428 (3)	
C50	0.17969 (6)	0.79661 (18)	0.08434 (5)	0.0417 (3)	
C51	0.23651 (6)	0.8059 (2)	0.07907 (5)	0.0524 (4)	
H51	0.2557	0.7051	0.0733	0.063*	
C52	0.26507 (7)	0.9625 (2)	0.08230 (6)	0.0642 (4)	
H52	0.3040	0.9689	0.0790	0.077*	
C53	0.23724 (8)	1.1088 (2)	0.09031 (7)	0.0671 (5)	
H53	0.2568	1.2158	0.0920	0.080*	
C54	0.18128 (7)	1.1013 (2)	0.09587 (7)	0.0630 (4)	
H54	0.1624	1.2028	0.1016	0.076*	
C55	0.15267 (6)	0.94555 (18)	0.09310 (6)	0.0499 (3)	
H55	0.1142	0.9403	0.0972	0.060*	
C56	−0.02987 (7)	0.7708 (2)	−0.01110 (6)	0.0648 (4)	
H56A	−0.0303	0.8070	−0.0463	0.078*	
H56B	−0.0420	0.8666	0.0063	0.078*	
H56C	−0.0588	0.6763	−0.0159	0.078*	
C57	0.29362 (8)	0.4032 (2)	0.25505 (7)	0.0727 (5)	
H57A	0.3319	0.3647	0.2831	0.087*	
H57B	0.2983	0.5215	0.2454	0.087*	
H57C	0.2842	0.3305	0.2232	0.087*	

### Atomic displacement parameters (Å^2^)

**Table:**

	*U*^11^	*U*^22^	*U*^33^	*U*^12^	*U*^13^	*U*^23^
O1	0.0487 (5)	0.0714 (7)	0.0598 (6)	0.0031 (5)	0.0247 (5)	0.0035 (5)
O2	0.0533 (6)	0.1092 (10)	0.0484 (6)	0.0056 (6)	0.0124 (5)	−0.0032 (6)
O3	0.0721 (7)	0.0479 (6)	0.0583 (6)	−0.0100 (5)	0.0363 (5)	−0.0031 (5)
O4	0.0544 (6)	0.0787 (8)	0.0639 (6)	0.0019 (5)	0.0287 (5)	0.0013 (6)
O5	0.0777 (7)	0.0800 (8)	0.0522 (6)	0.0030 (6)	0.0344 (6)	0.0090 (6)
O6	0.0835 (8)	0.0590 (7)	0.0465 (6)	−0.0073 (6)	0.0203 (5)	−0.0127 (5)
O7	0.0461 (5)	0.0767 (7)	0.0457 (5)	−0.0034 (5)	0.0116 (4)	0.0116 (5)
O8	0.0720 (7)	0.0703 (8)	0.0495 (6)	0.0015 (6)	0.0104 (5)	0.0125 (5)
O9	0.0827 (8)	0.0509 (6)	0.0801 (8)	−0.0027 (5)	0.0526 (6)	−0.0072 (5)
C1	0.0504 (7)	0.0462 (8)	0.0402 (7)	−0.0091 (6)	0.0225 (6)	−0.0065 (6)
C2	0.0511 (8)	0.0472 (8)	0.0488 (7)	−0.0079 (6)	0.0237 (6)	−0.0081 (6)
C3	0.0575 (8)	0.0567 (9)	0.0569 (8)	−0.0119 (7)	0.0346 (7)	−0.0090 (7)
C4	0.0703 (9)	0.0570 (9)	0.0489 (8)	−0.0145 (8)	0.0348 (7)	−0.0076 (7)
C5	0.0639 (9)	0.0511 (8)	0.0395 (7)	−0.0125 (7)	0.0254 (6)	−0.0079 (6)
C6	0.0751 (10)	0.0656 (10)	0.0397 (7)	−0.0108 (8)	0.0240 (7)	−0.0027 (7)
C7	0.0701 (10)	0.0741 (11)	0.0396 (8)	−0.0030 (8)	0.0132 (7)	−0.0014 (7)
C8	0.0520 (8)	0.0707 (11)	0.0409 (7)	−0.0041 (7)	0.0140 (6)	−0.0093 (7)
C9	0.0531 (8)	0.0620 (9)	0.0370 (7)	−0.0088 (7)	0.0188 (6)	−0.0072 (6)
C10	0.0533 (7)	0.0458 (8)	0.0382 (7)	−0.0092 (6)	0.0202 (6)	−0.0073 (6)
C11	0.0419 (7)	0.0491 (9)	0.0425 (7)	−0.0060 (6)	0.0179 (5)	−0.0005 (6)
C12	0.0404 (6)	0.0502 (8)	0.0387 (6)	−0.0053 (6)	0.0164 (5)	−0.0022 (6)
C13	0.0560 (8)	0.0564 (9)	0.0421 (7)	−0.0008 (7)	0.0213 (6)	0.0018 (6)
C14	0.0792 (11)	0.0728 (12)	0.0428 (8)	0.0048 (9)	0.0283 (7)	−0.0024 (7)
C15	0.0831 (12)	0.0667 (11)	0.0522 (9)	0.0063 (9)	0.0253 (8)	−0.0147 (8)
C16	0.0734 (10)	0.0526 (9)	0.0558 (9)	−0.0025 (8)	0.0214 (8)	−0.0074 (7)
C17	0.0562 (8)	0.0514 (9)	0.0431 (7)	−0.0076 (7)	0.0189 (6)	−0.0034 (6)
C18	0.0481 (8)	0.0660 (11)	0.0800 (11)	−0.0018 (7)	0.0281 (8)	−0.0105 (9)
C19	0.0503 (9)	0.1288 (18)	0.0647 (11)	0.0001 (10)	0.0235 (8)	−0.0012 (11)
C20	0.0524 (8)	0.0443 (8)	0.0428 (7)	0.0064 (6)	0.0161 (6)	−0.0046 (6)
C21	0.0538 (8)	0.0541 (9)	0.0543 (8)	0.0066 (7)	0.0190 (7)	−0.0057 (7)
C22	0.0522 (9)	0.0711 (11)	0.0680 (10)	0.0111 (8)	0.0152 (8)	−0.0005 (9)
C23	0.0637 (10)	0.0743 (12)	0.0596 (10)	0.0198 (9)	0.0061 (8)	0.0057 (9)
C24	0.0661 (10)	0.0587 (10)	0.0458 (8)	0.0139 (8)	0.0129 (7)	0.0032 (7)
C25	0.0838 (12)	0.0798 (12)	0.0462 (9)	0.0191 (10)	0.0138 (8)	0.0158 (8)
C26	0.0922 (13)	0.0774 (12)	0.0470 (9)	0.0135 (10)	0.0282 (9)	0.0156 (8)
C27	0.0720 (10)	0.0560 (9)	0.0456 (8)	0.0060 (7)	0.0277 (7)	0.0013 (7)
C28	0.0609 (8)	0.0468 (8)	0.0374 (7)	0.0079 (6)	0.0180 (6)	0.0001 (6)
C29	0.0592 (8)	0.0422 (8)	0.0408 (7)	0.0087 (6)	0.0166 (6)	−0.0028 (6)
C30	0.0499 (7)	0.0499 (8)	0.0378 (7)	−0.0040 (6)	0.0206 (6)	−0.0053 (6)
C31	0.0389 (6)	0.0499 (8)	0.0421 (7)	−0.0024 (6)	0.0171 (5)	0.0015 (6)
C32	0.0514 (8)	0.0688 (11)	0.0477 (8)	−0.0051 (7)	0.0127 (6)	0.0090 (7)
C33	0.0592 (10)	0.0759 (13)	0.0786 (12)	0.0030 (9)	0.0159 (9)	0.0323 (10)
C34	0.0591 (10)	0.0533 (10)	0.1152 (16)	0.0061 (8)	0.0377 (10)	0.0239 (11)
C35	0.0583 (9)	0.0487 (9)	0.0877 (12)	−0.0038 (7)	0.0370 (9)	−0.0069 (8)
C36	0.0449 (7)	0.0497 (8)	0.0508 (8)	−0.0018 (6)	0.0211 (6)	−0.0027 (6)
C37	0.0543 (9)	0.0845 (13)	0.0775 (11)	−0.0077 (8)	0.0322 (8)	−0.0207 (10)
C38	0.0677 (10)	0.0766 (12)	0.0564 (9)	−0.0061 (9)	0.0281 (8)	−0.0016 (8)
C39	0.0455 (7)	0.0409 (7)	0.0420 (7)	−0.0058 (6)	0.0193 (6)	−0.0018 (6)
C40	0.0468 (7)	0.0502 (8)	0.0459 (7)	−0.0070 (6)	0.0172 (6)	0.0025 (6)
C41	0.0433 (7)	0.0673 (10)	0.0611 (9)	−0.0073 (7)	0.0204 (7)	0.0036 (8)
C42	0.0542 (8)	0.0655 (10)	0.0661 (10)	−0.0084 (7)	0.0351 (8)	0.0009 (8)
C43	0.0573 (8)	0.0469 (8)	0.0495 (8)	−0.0102 (6)	0.0285 (7)	−0.0028 (6)
C44	0.0760 (10)	0.0571 (9)	0.0508 (8)	−0.0134 (8)	0.0364 (8)	−0.0038 (7)
C45	0.0855 (11)	0.0553 (9)	0.0404 (7)	−0.0115 (8)	0.0271 (8)	0.0007 (7)
C46	0.0650 (9)	0.0437 (8)	0.0433 (7)	−0.0045 (7)	0.0150 (7)	0.0012 (6)
C47	0.0527 (8)	0.0430 (8)	0.0437 (7)	−0.0033 (6)	0.0199 (6)	−0.0006 (6)
C48	0.0527 (7)	0.0361 (7)	0.0416 (7)	−0.0069 (6)	0.0212 (6)	−0.0028 (5)
C49	0.0478 (7)	0.0462 (8)	0.0359 (6)	−0.0005 (6)	0.0173 (6)	0.0015 (6)
C50	0.0425 (7)	0.0495 (8)	0.0319 (6)	−0.0033 (6)	0.0125 (5)	0.0035 (5)
C51	0.0465 (7)	0.0678 (10)	0.0445 (7)	−0.0055 (7)	0.0187 (6)	0.0017 (7)
C52	0.0512 (8)	0.0851 (13)	0.0567 (9)	−0.0212 (9)	0.0205 (7)	0.0038 (8)
C53	0.0659 (10)	0.0604 (11)	0.0662 (10)	−0.0209 (8)	0.0144 (8)	0.0093 (8)
C54	0.0619 (9)	0.0480 (9)	0.0704 (10)	−0.0062 (7)	0.0145 (8)	0.0047 (8)
C55	0.0464 (7)	0.0493 (8)	0.0507 (8)	−0.0028 (6)	0.0141 (6)	0.0026 (6)
C56	0.0522 (9)	0.0776 (12)	0.0547 (9)	−0.0015 (8)	0.0081 (7)	0.0095 (8)
C57	0.0609 (10)	0.0713 (12)	0.0724 (11)	0.0022 (8)	0.0089 (8)	0.0193 (9)

### Geometric parameters (Å, °)

**Table:**

O1—C2	1.3671 (17)	C25—C26	1.352 (3)
O1—C18	1.4257 (17)	C25—H25	0.9500
O2—C8	1.3650 (18)	C26—C27	1.414 (2)
O2—C19	1.425 (2)	C26—H26	0.9500
O3—C11	1.2196 (17)	C27—C28	1.362 (2)
O4—C21	1.3677 (18)	C28—C29	1.422 (2)
O4—C37	1.4288 (18)	C28—H28	0.9500
O5—C27	1.3718 (19)	C30—C31	1.477 (2)
O5—C38	1.4154 (19)	C31—C36	1.3902 (19)
O6—C30	1.2172 (16)	C31—C32	1.3957 (19)
O7—C40	1.3653 (16)	C32—C33	1.373 (2)
O7—C56	1.4298 (17)	C32—H32	0.9500
O8—C46	1.3656 (18)	C33—C34	1.373 (3)
O8—C57	1.420 (2)	C33—H33	0.9500
O9—C49	1.2178 (16)	C34—C35	1.383 (3)
C1—C2	1.3773 (18)	C34—H34	0.9500
C1—C10	1.4125 (19)	C35—C36	1.383 (2)
C1—C11	1.5069 (17)	C35—H35	0.9500
C2—C3	1.4097 (19)	C36—H36	0.9500
C3—C4	1.363 (2)	C37—H37A	0.9800
C3—H3	0.9500	C37—H37B	0.9800
C4—C5	1.411 (2)	C37—H37C	0.9800
C4—H4	0.9500	C38—H38A	0.9800
C5—C6	1.412 (2)	C38—H38B	0.9800
C5—C10	1.4260 (18)	C38—H38C	0.9800
C6—C7	1.351 (2)	C39—C40	1.3780 (19)
C6—H6	0.9500	C39—C48	1.4198 (18)
C7—C8	1.410 (2)	C39—C49	1.5034 (17)
C7—H7	0.9500	C40—C41	1.4101 (19)
C8—C9	1.367 (2)	C41—C42	1.361 (2)
C9—C10	1.4285 (19)	C41—H41	0.9500
C9—H9	0.9500	C42—C43	1.407 (2)
C11—C12	1.4916 (18)	C42—H42	0.9500
C12—C17	1.388 (2)	C43—C44	1.422 (2)
C12—C13	1.3904 (18)	C43—C48	1.4225 (18)
C13—C14	1.377 (2)	C44—C45	1.354 (2)
C13—H13	0.9500	C44—H44	0.9500
C14—C15	1.378 (2)	C45—C46	1.411 (2)
C14—H14	0.9500	C45—H45	0.9500
C15—C16	1.377 (2)	C46—C47	1.3659 (19)
C15—H15	0.9500	C47—C48	1.4194 (19)
C16—C17	1.385 (2)	C47—H47	0.9500
C16—H16	0.9500	C49—C50	1.4800 (19)
C17—H17	0.9500	C50—C55	1.389 (2)
C18—H18A	0.9800	C50—C51	1.3919 (18)
C18—H18B	0.9800	C51—C52	1.386 (2)
C18—H18C	0.9800	C51—H51	0.9500
C19—H19A	0.9800	C52—C53	1.375 (3)
C19—H19B	0.9800	C52—H52	0.9500
C19—H19C	0.9800	C53—C54	1.375 (2)
C20—C21	1.378 (2)	C53—H53	0.9500
C20—C29	1.420 (2)	C54—C55	1.382 (2)
C20—C30	1.5047 (18)	C54—H54	0.9500
C21—C22	1.405 (2)	C55—H55	0.9500
C22—C23	1.361 (3)	C56—H56A	0.9800
C22—H22	0.9500	C56—H56B	0.9800
C23—C24	1.414 (2)	C56—H56C	0.9800
C23—H23	0.9500	C57—H57A	0.9800
C24—C25	1.415 (2)	C57—H57B	0.9800
C24—C29	1.422 (2)	C57—H57C	0.9800
			
C2—O1—C18	118.35 (12)	C28—C29—C24	119.08 (13)
C8—O2—C19	117.38 (12)	O6—C30—C31	121.36 (13)
C21—O4—C37	118.51 (13)	O6—C30—C20	119.53 (13)
C27—O5—C38	117.56 (11)	C31—C30—C20	119.10 (11)
C40—O7—C56	117.87 (11)	C36—C31—C32	119.58 (14)
C46—O8—C57	116.82 (12)	C36—C31—C30	121.25 (12)
C2—C1—C10	120.29 (12)	C32—C31—C30	119.17 (13)
C2—C1—C11	118.21 (12)	C33—C32—C31	119.91 (16)
C10—C1—C11	121.50 (12)	C33—C32—H32	120.0
O1—C2—C1	115.43 (12)	C31—C32—H32	120.0
O1—C2—C3	123.71 (13)	C32—C33—C34	120.42 (17)
C1—C2—C3	120.86 (13)	C32—C33—H33	119.8
C4—C3—C2	119.47 (13)	C34—C33—H33	119.8
C4—C3—H3	120.3	C33—C34—C35	120.31 (17)
C2—C3—H3	120.3	C33—C34—H34	119.8
C3—C4—C5	121.72 (13)	C35—C34—H34	119.8
C3—C4—H4	119.1	C34—C35—C36	119.97 (17)
C5—C4—H4	119.1	C34—C35—H35	120.0
C4—C5—C6	122.46 (13)	C36—C35—H35	120.0
C4—C5—C10	118.65 (13)	C35—C36—C31	119.81 (14)
C6—C5—C10	118.88 (13)	C35—C36—H36	120.1
C7—C6—C5	121.10 (14)	C31—C36—H36	120.1
C7—C6—H6	119.5	O4—C37—H37A	109.5
C5—C6—H6	119.5	O4—C37—H37B	109.5
C6—C7—C8	120.54 (15)	H37A—C37—H37B	109.5
C6—C7—H7	119.7	O4—C37—H37C	109.5
C8—C7—H7	119.7	H37A—C37—H37C	109.5
O2—C8—C9	124.95 (14)	H37B—C37—H37C	109.5
O2—C8—C7	114.27 (14)	O5—C38—H38A	109.5
C9—C8—C7	120.78 (14)	O5—C38—H38B	109.5
C8—C9—C10	119.92 (13)	H38A—C38—H38B	109.5
C8—C9—H9	120.0	O5—C38—H38C	109.5
C10—C9—H9	120.0	H38A—C38—H38C	109.5
C1—C10—C5	118.98 (13)	H38B—C38—H38C	109.5
C1—C10—C9	122.27 (12)	C40—C39—C48	120.48 (12)
C5—C10—C9	118.75 (13)	C40—C39—C49	120.95 (12)
O3—C11—C12	121.24 (12)	C48—C39—C49	118.56 (11)
O3—C11—C1	120.24 (12)	O7—C40—C39	115.54 (12)
C12—C11—C1	118.51 (12)	O7—C40—C41	123.97 (12)
C17—C12—C13	119.17 (13)	C39—C40—C41	120.49 (13)
C17—C12—C11	121.52 (12)	C42—C41—C40	119.61 (14)
C13—C12—C11	119.29 (13)	C42—C41—H41	120.2
C14—C13—C12	120.03 (15)	C40—C41—H41	120.2
C14—C13—H13	120.0	C41—C42—C43	121.94 (13)
C12—C13—H13	120.0	C41—C42—H42	119.0
C13—C14—C15	120.22 (15)	C43—C42—H42	119.0
C13—C14—H14	119.9	C42—C43—C44	123.02 (13)
C15—C14—H14	119.9	C42—C43—C48	118.77 (13)
C16—C15—C14	120.62 (15)	C44—C43—C48	118.21 (14)
C16—C15—H15	119.7	C45—C44—C43	121.32 (14)
C14—C15—H15	119.7	C45—C44—H44	119.3
C15—C16—C17	119.28 (16)	C43—C44—H44	119.3
C15—C16—H16	120.4	C44—C45—C46	120.20 (13)
C17—C16—H16	120.4	C44—C45—H45	119.9
C16—C17—C12	120.67 (14)	C46—C45—H45	119.9
C16—C17—H17	119.7	O8—C46—C47	124.56 (14)
C12—C17—H17	119.7	O8—C46—C45	114.65 (13)
O1—C18—H18A	109.5	C47—C46—C45	120.78 (14)
O1—C18—H18B	109.5	C46—C47—C48	120.08 (13)
H18A—C18—H18B	109.5	C46—C47—H47	120.0
O1—C18—H18C	109.5	C48—C47—H47	120.0
H18A—C18—H18C	109.5	C47—C48—C39	121.91 (12)
H18B—C18—H18C	109.5	C47—C48—C43	119.39 (12)
O2—C19—H19A	109.5	C39—C48—C43	118.68 (12)
O2—C19—H19B	109.5	O9—C49—C50	121.35 (12)
H19A—C19—H19B	109.5	O9—C49—C39	119.85 (12)
O2—C19—H19C	109.5	C50—C49—C39	118.67 (11)
H19A—C19—H19C	109.5	C55—C50—C51	119.09 (13)
H19B—C19—H19C	109.5	C55—C50—C49	121.53 (12)
C21—C20—C29	120.70 (13)	C51—C50—C49	119.37 (13)
C21—C20—C30	117.90 (13)	C52—C51—C50	119.87 (15)
C29—C20—C30	121.38 (12)	C52—C51—H51	120.1
O4—C21—C20	115.41 (13)	C50—C51—H51	120.1
O4—C21—C22	123.77 (14)	C53—C52—C51	120.18 (15)
C20—C21—C22	120.82 (15)	C53—C52—H52	119.9
C23—C22—C21	119.31 (16)	C51—C52—H52	119.9
C23—C22—H22	120.3	C52—C53—C54	120.55 (15)
C21—C22—H22	120.3	C52—C53—H53	119.7
C22—C23—C24	122.01 (15)	C54—C53—H53	119.7
C22—C23—H23	119.0	C53—C54—C55	119.67 (16)
C24—C23—H23	119.0	C53—C54—H54	120.2
C23—C24—C25	122.93 (15)	C55—C54—H54	120.2
C23—C24—C29	118.85 (15)	C54—C55—C50	120.63 (14)
C25—C24—C29	118.21 (15)	C54—C55—H55	119.7
C26—C25—C24	121.94 (15)	C50—C55—H55	119.7
C26—C25—H25	119.0	O7—C56—H56A	109.5
C24—C25—H25	119.0	O7—C56—H56B	109.5
C25—C26—C27	119.62 (15)	H56A—C56—H56B	109.5
C25—C26—H26	120.2	O7—C56—H56C	109.5
C27—C26—H26	120.2	H56A—C56—H56C	109.5
C28—C27—O5	124.96 (14)	H56B—C56—H56C	109.5
C28—C27—C26	120.91 (16)	O8—C57—H57A	109.5
O5—C27—C26	114.13 (14)	O8—C57—H57B	109.5
C27—C28—C29	120.23 (13)	H57A—C57—H57B	109.5
C27—C28—H28	119.9	O8—C57—H57C	109.5
C29—C28—H28	119.9	H57A—C57—H57C	109.5
C20—C29—C28	122.64 (12)	H57B—C57—H57C	109.5
C20—C29—C24	118.28 (14)		
			
C18—O1—C2—C1	−173.65 (13)	C30—C20—C29—C24	176.62 (13)
C18—O1—C2—C3	6.5 (2)	C27—C28—C29—C20	179.41 (14)
C10—C1—C2—O1	178.93 (12)	C27—C28—C29—C24	−0.3 (2)
C11—C1—C2—O1	−1.35 (19)	C23—C24—C29—C20	0.2 (2)
C10—C1—C2—C3	−1.3 (2)	C25—C24—C29—C20	−179.92 (15)
C11—C1—C2—C3	178.45 (13)	C23—C24—C29—C28	179.91 (14)
O1—C2—C3—C4	−178.48 (14)	C25—C24—C29—C28	−0.2 (2)
C1—C2—C3—C4	1.7 (2)	C21—C20—C30—O6	90.52 (17)
C2—C3—C4—C5	−0.5 (2)	C29—C20—C30—O6	−87.62 (17)
C3—C4—C5—C6	177.90 (15)	C21—C20—C30—C31	−88.33 (16)
C3—C4—C5—C10	−1.2 (2)	C29—C20—C30—C31	93.53 (16)
C4—C5—C6—C7	179.86 (15)	O6—C30—C31—C36	170.52 (13)
C10—C5—C6—C7	−1.1 (2)	C20—C30—C31—C36	−10.65 (18)
C5—C6—C7—C8	−0.6 (3)	O6—C30—C31—C32	−9.4 (2)
C19—O2—C8—C9	2.8 (2)	C20—C30—C31—C32	169.46 (12)
C19—O2—C8—C7	−177.25 (16)	C36—C31—C32—C33	0.5 (2)
C6—C7—C8—O2	−178.67 (15)	C30—C31—C32—C33	−179.62 (14)
C6—C7—C8—C9	1.3 (3)	C31—C32—C33—C34	−0.8 (2)
O2—C8—C9—C10	179.67 (14)	C32—C33—C34—C35	0.6 (3)
C7—C8—C9—C10	−0.3 (2)	C33—C34—C35—C36	0.0 (2)
C2—C1—C10—C5	−0.4 (2)	C34—C35—C36—C31	−0.4 (2)
C11—C1—C10—C5	179.88 (12)	C32—C31—C36—C35	0.1 (2)
C2—C1—C10—C9	−179.93 (13)	C30—C31—C36—C35	−179.77 (12)
C11—C1—C10—C9	0.4 (2)	C56—O7—C40—C39	−178.58 (13)
C4—C5—C10—C1	1.6 (2)	C56—O7—C40—C41	1.2 (2)
C6—C5—C10—C1	−177.49 (13)	C48—C39—C40—O7	−179.37 (12)
C4—C5—C10—C9	−178.87 (13)	C49—C39—C40—O7	1.99 (19)
C6—C5—C10—C9	2.0 (2)	C48—C39—C40—C41	0.9 (2)
C8—C9—C10—C1	178.14 (14)	C49—C39—C40—C41	−177.78 (13)
C8—C9—C10—C5	−1.4 (2)	O7—C40—C41—C42	−179.41 (15)
C2—C1—C11—O3	−77.82 (17)	C39—C40—C41—C42	0.3 (2)
C10—C1—C11—O3	101.89 (16)	C40—C41—C42—C43	−0.2 (2)
C2—C1—C11—C12	101.28 (15)	C41—C42—C43—C44	178.24 (15)
C10—C1—C11—C12	−79.00 (16)	C41—C42—C43—C48	−1.0 (2)
O3—C11—C12—C17	−173.34 (13)	C42—C43—C44—C45	−179.64 (15)
C1—C11—C12—C17	7.57 (18)	C48—C43—C44—C45	−0.4 (2)
O3—C11—C12—C13	8.05 (19)	C43—C44—C45—C46	−1.0 (2)
C1—C11—C12—C13	−171.04 (12)	C57—O8—C46—C47	2.4 (2)
C17—C12—C13—C14	−0.7 (2)	C57—O8—C46—C45	−176.83 (14)
C11—C12—C13—C14	177.96 (13)	C44—C45—C46—O8	−179.35 (14)
C12—C13—C14—C15	0.8 (2)	C44—C45—C46—C47	1.4 (2)
C13—C14—C15—C16	−0.1 (3)	O8—C46—C47—C48	−179.64 (13)
C14—C15—C16—C17	−0.7 (3)	C45—C46—C47—C48	−0.5 (2)
C15—C16—C17—C12	0.8 (2)	C46—C47—C48—C39	177.48 (13)
C13—C12—C17—C16	−0.1 (2)	C46—C47—C48—C43	−0.9 (2)
C11—C12—C17—C16	−178.70 (13)	C40—C39—C48—C47	179.53 (13)
C37—O4—C21—C20	−174.38 (13)	C49—C39—C48—C47	−1.80 (19)
C37—O4—C21—C22	5.6 (2)	C40—C39—C48—C43	−2.12 (19)
C29—C20—C21—O4	−178.09 (13)	C49—C39—C48—C43	176.55 (12)
C30—C20—C21—O4	3.8 (2)	C42—C43—C48—C47	−179.42 (13)
C29—C20—C21—C22	2.0 (2)	C44—C43—C48—C47	1.3 (2)
C30—C20—C21—C22	−176.19 (14)	C42—C43—C48—C39	2.2 (2)
O4—C21—C22—C23	178.90 (16)	C44—C43—C48—C39	−177.13 (13)
C20—C21—C22—C23	−1.2 (3)	C40—C39—C49—O9	−101.93 (16)
C21—C22—C23—C24	−0.1 (3)	C48—C39—C49—O9	79.40 (17)
C22—C23—C24—C25	−179.28 (18)	C40—C39—C49—C50	82.13 (16)
C22—C23—C24—C29	0.5 (3)	C48—C39—C49—C50	−96.53 (15)
C23—C24—C25—C26	179.99 (18)	O9—C49—C50—C55	167.01 (13)
C29—C24—C25—C26	0.2 (3)	C39—C49—C50—C55	−17.12 (18)
C24—C25—C26—C27	0.4 (3)	O9—C49—C50—C51	−13.91 (19)
C38—O5—C27—C28	7.5 (2)	C39—C49—C50—C51	161.96 (12)
C38—O5—C27—C26	−171.85 (15)	C55—C50—C51—C52	−0.36 (19)
C25—C26—C27—C28	−0.9 (3)	C49—C50—C51—C52	−179.46 (13)
C25—C26—C27—O5	178.48 (16)	C50—C51—C52—C53	−0.5 (2)
O5—C27—C28—C29	−178.51 (14)	C51—C52—C53—C54	0.8 (2)
C26—C27—C28—C29	0.8 (2)	C52—C53—C54—C55	−0.3 (2)
C21—C20—C29—C28	178.86 (13)	C53—C54—C55—C50	−0.5 (2)
C30—C20—C29—C28	−3.0 (2)	C51—C50—C55—C54	0.8 (2)
C21—C20—C29—C24	−1.5 (2)	C49—C50—C55—C54	179.92 (13)

### Hydrogen-bond geometry (Å, °)

**Table:**

*D*—H···*A*	*D*—H	H···*A*	*D*···*A*	*D*—H···*A*
C36—H36···O2	0.95	2.56	3.4116 (18)	149
C56—H56A···O5^i^	0.98	2.54	3.4862 (19)	161
C52—H52···O3^i^	0.95	2.46	3.395 (2)	168
C34—H34···O6^ii^	0.95	2.41	3.143 (2)	133
C54—H54···O9^ii^	0.95	2.58	3.2451 (19)	128
C19—H19B···O9^iii^	0.98	2.59	3.116 (2)	113

Symmetry codes:  (i) *x*, *y*+1, *z*−1; (ii) *x*, *y*+1, *z*; (iii) *x*, *y*, *z*+1.
